# Electrophoretic Co-deposition of Polyetheretherketone and Graphite Particles: Microstructure, Electrochemical Corrosion Resistance, and Coating Adhesion to a Titanium Alloy

**DOI:** 10.3390/ma13153251

**Published:** 2020-07-22

**Authors:** Aleksandra Fiołek, Sławomir Zimowski, Agnieszka Kopia, Alicja Łukaszczyk, Tomasz Moskalewicz

**Affiliations:** 1 Faculty of Metals Engineering and Industrial Computer Science, AGH University of Science and Technology, Czarnowiejska 66, 30-054 Kraków, Poland; kopia@agh.edu.pl; 2 Faculty of Mechanical Engineering and Robotics, AGH University of Science and Technology, Mickiewicza Av. 30, 30-059 Kraków, Poland; zimowski@agh.edu.pl; 3 Faculty of Foundry Engineering, AGH University of Science and Technology, Reymonta 23, 30-059 Kraków, Poland; alicjal@agh.edu.pl

**Keywords:** graphite, polyetheretherketone, composite coating, electrophoretic deposition, microstructure

## Abstract

The present study explores the possibilities of fabricating a graphite/polyetheretherketone (PEEK) composite coating on a Ti-6Al-4V titanium alloy through duplex treatment consisting of electrophoretic deposition (EPD) and heat treatment. It has been found that the electrophoretic co-deposition of graphite and PEEK microparticles can be performed from environmentally-friendly pure ethanolic suspensions. Zeta potential measurements and a study of the interaction between both particle types with the use of transmission electron microscopy allowed potential mechanisms of particle co-deposition to be indicated. Microstructure characterization was performed on macro-, micro- and nanoscale using visible light microscopy, X-ray diffractometry and electron microscopy. This allowed the coating homogeneity and distribution of graphite particles in the polymer matrix to be described. Graphite particles in the form of graphene nanosheet packages were relatively evenly distributed in the coating matrix and oriented parallel to the coating surface. The heat-treated coatings showed high scratch resistance and no adhesive type destruction was observed, but they were highly susceptible to deformation. The corrosion measurements were performed with use of electrochemical techniques like open circuit potential and linear sweep voltamperometry. The coated alloy indicated better electrochemical corrosion resistance compared with the uncoated alloy. This work showed the high versatility of the electrophoretic co-deposition of graphite and PEEK particles, which combined with post-EPD heat treatment allows composite coatings to be fabricated with controlled distribution of graphite particles.

## 1. Introduction

Polyetheretherketone (PEEK) is a high-performance thermoplastic polymer, with an amorphous or semi-crystalline structure depending on the applied processing and heat treatment route [1,2]. This material exhibits high thermal stability, easy processing and high chemical resistance. In addition, it combines low density with good mechanical and tribological properties, such as high mechanical strength and elastic modulus, as well as wear resistance [3,4,5]. The introduction of a reinforcement phase into the PEEK matrix may result in unique properties being given to the final material, therefore PEEK is often used as the matrix for various types of composites. For example, Baştan et al. [6] introduced hydroxyapatite (HA) microparticles into the PEEK matrix to increase bioactivity. Vail et al. [7] used the polytetrafluoroethylene (PTFE) filaments in the PEEK matrix to reduce the coefficient of friction (COF) and wear rate.

PEEK may be used as a matrix for bulk composites, but also as a matrix of composite coatings improving various properties of metallic materials. In recent years, PEEK and PEEK-based coatings have been produced by various methods, such as thermal spraying [8] and printing technology [9]. Another method that allows the production of this type of coating is electrophoretic deposition (EPD). This is a method in which there is fast-growing interest due to the short time of coating production, uncomplicated and inexpensive equipment as well as great flexibility in the selection of materials for deposition [10].

Due to the unique properties of PEEK, the EPD of both PEEK [11,12,13,14] and PEEK-based [6,15,16,17,18,19] composite coatings has been widely studied, also with respect to improving the tribological properties of metallic materials. In our previous works, we have shown that it is possible to fabricate repeatable and homogeneous, semi-crystalline or amorphous PEEK [20] and composite PEEK-based coatings [21,22,23,24,25] using EPD and post-EPD heat treatment. However, despite the fact that these coatings improved the wear resistance of titanium alloys, the reduction of the coefficient of friction was not at a satisfactory level, usually about 0.3 in cooperation with an alumina ball. Therefore, the challenge is to develop wear resistant coatings with low a COF, at the level of 0.1 or below this value. It should be emphasized that the development of low-friction PEEK-based coatings, with the exception of the MoS_2_/PEEK708 coating [25], is missing in the available literature. In those works, also the kinetics and mechanism of the electrophoretic co-deposition of PEEK particles with oxides [21], nitrides [22,23,24] and sulfides [25] were investigated. It was found that only Al_2_O_3_ [21] and PEEK [20] particles were successfully deposited by anaphoresis from a suspension containing only ethanol as a dispersion phase. In the case of the co-deposition of PEEK with Si_3_N_4_ [22,23] or TiN [24] nanoparticles or MoS_2_ [25] nanosheets, it was necessary to additionally stabilize the suspension by using cationic chitosan polyelectrolyte or polyethylenimine (PEI). Although this addition allowed the cathodic co-deposition of polymer microparticles with nitride or sulfide nanoparticles, the degradation of chitosan during heat treatment caused the formation of porosity in the coatings, which resulted in a decrease in the subsequent scratch resistance and tribological properties. In particular, in terms of tribological properties, it is important that the coating is as uniform and devoid of surface irregularity as possible. No less important is the selection of the appropriate reinforcement phase, which, in combination with the matrix, gives unique properties to the composite. For example, it is possible to significantly reduce the coefficient of friction by using a material with a self-lubricating capacity [26]. Graphite is one of the allotropic forms of carbon and, next to molybdenum disulfide, is the most commonly used solid lubricant. Due to its hexagonal structure with the axial ratio c/a in the elementary cell of about 2.7 (where a and c are parameters of the elementary cell), it exhibits strong anisotropy of thermal and electrical properties. Graphite consists of layers of carbon atoms connected by strong covalent bonds, but between the layers there are weak van der Waals forces. Individual layers can be easily moved relative to each other, therefore, graphite has excellent lubrication properties [27,28]. The individual two-dimensional one-atom-thick layers of graphite are called graphene. It is characterized by low density, high mechanical strength, high surface area and high chemical stability [29,30,31,32]. Due to its outstanding properties, graphene is extremely attractive from a mechanical and tribological point of view [33]. Graphene has already been used as a filler in the composites of various polymers, for example, polyimide [34], polytetrafluorethylene [35] or polyacrylonitrile [36]. In recent years, several papers have also been published describing the production and properties of graphene/PEEK composites [37,38,39,40,41,42,43]. The EPD of pure graphene has also been reported in the literature and the authors of those works proved that it is possible to obtain graphene coatings on various substrates, e.g., silicon [44] or titanium alloy [32]. However, as far as we know, the present paper is the first study regarding the electrophoretic deposition of composite PEEK-based coatings incorporating graphite particles consisting of many layers of graphene stacked together. The aim of the present work is to develop deposition conditions and investigate the EPD mechanism, as well as the heat treatment process, for the fabrication of novel nanocomposite graphite/PEEK 708 coatings on Ti-6Al-4V alloy substrates. It is also particularly interesting to compare the EPD process and mechanism of the co-deposition of PEEK and graphite particles with respect to the co-deposition of PEEK with oxides, nitrides and sulfides.

## 2. Materials and Methods

Graphite particles in the form of graphene nanosheet stacks and PEEK 708 (Victrex Europa GmbH, Hofheim am Taunus, Germany) microparticles were used as composite coating components. Graphite was delivered by Nanostructured and Amorphous Materials, Inc. (Houston, TX, USA). According to the manufacturer, the particle diameter was in the range of 5–10 μm, while the thickness was from 4 nm to 20 nm. PEEK 708 has a melting point of 374 °C, a density of 1.32 g/cm^3^ and a particle size of up to 10 μm. As a substrate for coating deposition, a two-phase (α + β) Ti-6Al-4V titanium alloy (BÖHLER Edelstahl GmbH, Düsseldorf, Germany) was used. The alloy in the form of a bar with a 22 mm diameter was cut into discs with a thickness of 3 mm. The surface of discs was ground using sandpaper with a gradually increasing gradation from 200 to 3000 and then polished using a Struers (Ballerup, Denmark) standard colloidal silicon suspension (OP-S, 0.04 μm). Directly before the deposition, the surface was washed with distilled water and degreased with technical ethanol.

The following suspensions were prepared for coating deposition: 1 g/L, 2 g/L or 4 g/L of graphite powder and 30 g/L of PEEK powder were mixed with pure ethanol (with a purity of 99.8 %), which was the dispersion medium. The prepared suspension was then magnetically stirred for 5 min and ultrasonically dispersed for 15 min to eliminate the particle agglomerates.

The zeta potential of graphite and PEEK particles as a function of the suspension’s pH was measured using the Laser Doppler Velocimetry (LDV) technique with a Zetasizer Nano ZS 90 of Malvern Instruments Ltd., (Malvern, UK). The pH value was increased with sodium hydroxide and lowered with citric acid, and its value was controlled with an ELMETRON CPC-505 pH-meter (Zabrze, Poland).

Direct current electrophoretic deposition was carried out in a standard two-electrode system using an EX752M PSU Multi-mode power supply (Huntingdon, UK). Austenitic stainless steel was used as a counter electrode (cathode), while the titanium alloy, on which the coating was deposited, was an anode. Constant voltage in the range of 10–100 V with 10 V changes and a constant time of 40 s were applied. The distance between electrodes was constant at 10 mm and the suspension was magnetically stirred (~ 50 rpm) throughout the deposition process to avoid particle sedimentation. As-deposited coatings were left for 1 h until they dried in atmospheric air.

The coated alloy was heat treated in a Carbolite-Gero LHT 4/30 (Derbyshire, UK) laboratory oven. The treatment consisted of heating samples with the furnace to a temperature of 390 °C at a constant rate of 4.5 °C/min, holding this temperature for 40 min, and then cooling with the furnace to room temperature (RT) with a cooling rate of 2 °C/min.

Investigations of the coating components and coating microstructure were carried out using an FEI Nova NanoSEM 450 (Eindhoven, the Netherlands) scanning electron microscope (SEM), JEOL JEM-2010 ARP microscope (Tokyo, Japan) and Tecnai TF 20 X-TWIN (FEI) (Eindhoven, the Netherlands) transmission electron microscopes (TEM). Thin foils for TEM investigation of graphite particles and the suspension used for EPD were obtained by placing a drop of the ethanol containing graphite particles or EPD suspension, respectively, on a copper grid and air-drying. The coating microstructure was investigated also on cross-section lamellae, that were cut by a focused ion beam (FIB) using an FEI QUANTA 3D 200i device (Eindhoven, the Netherlands). Phase analysis was conducted with the use of X-ray diffraction (XRD) in Bragg–Brentano geometry using a Panalytical Empyrean DY1061 diffractometer (Almelo, the Netherlands) and in TEM by selected area electron diffraction (SAED). The electron diffraction patterns and fast Fourier transformation (FFT) patterns were interpreted with the use of Java Electron Microscopy Software (JEMS, Pierre Stadelmann, Switzerland). Image analysis was performed using Gatan’s ‘Digital Micrograph’ (Pleasanton, CA, USA) software.

In order to determine a scratch resistance of the coatings the scratch tests were carried out using a Rockwell C diamond stylus with a radius of 0.2 mm with the use of Micro-Combi Tester (MCT) from CSM Instruments (Peuseux, Switzerland). During the tests the load increased linearly from 0.01 to 30 N on a scratch length of 5 mm with a constant sample velocity of 5 mm/min. In the scratch tests, the critical load (Lc) corresponding to the load at which some failure of the coating occurs, involving the load L_C1_ at which the first cohesive cracks appear or the load L_C2_ causing adhesive damage, was investigated. Additionally, the scratch tests with a constant load equal to 0.25 N at the same parameters (5 mm of the scratch length and 5 mm/min of the velocity) were performed to determine the COF of the coatings vs. a Rockwell C diamond stylus. The measurements were repeated three times for each sample at defined test parameters. The paper presents representative results of the performed tests.

A PGSTAT302 AUTOLAB potentiostat/galvanostat (Utrecht, the Netherlands) was used in corrosion resistance experiments. All potentials were measured vs. SCE (3M KCl solution) and the counter electrode was made of platinum plate. The measurements were performed using a classical three-electrode cell. NaCl (3.5 wt.%) aqueous solution was used as the electrolyte. Experiments were performed in aerated solutions at the temperature of 25 °C. The polarization curves were obtained with a scan rate of 1 mV/s.

## 3. Results and Discussion

**Figure 1:**
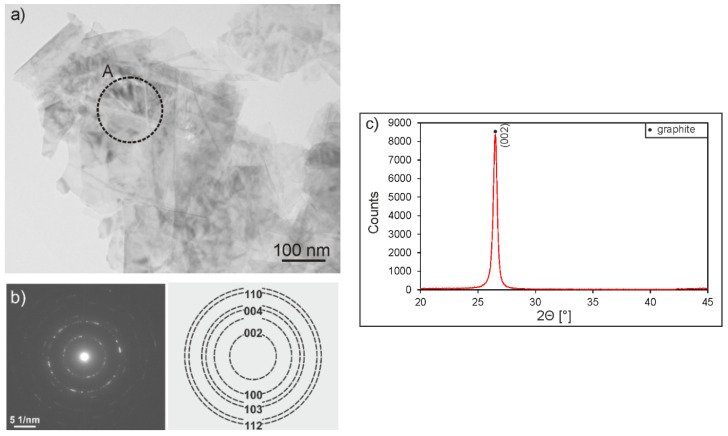
Transmission electron microscopy (TEM) micrograph (**a**) and selected area electron diffraction (SAED) pattern of the area marked as A in Figure 1a with identification (**b**), as well as the X-ray diffraction (XRD) pattern (**c**) of graphite particles consisting of graphene layers stacked together.

**Figure 2:**
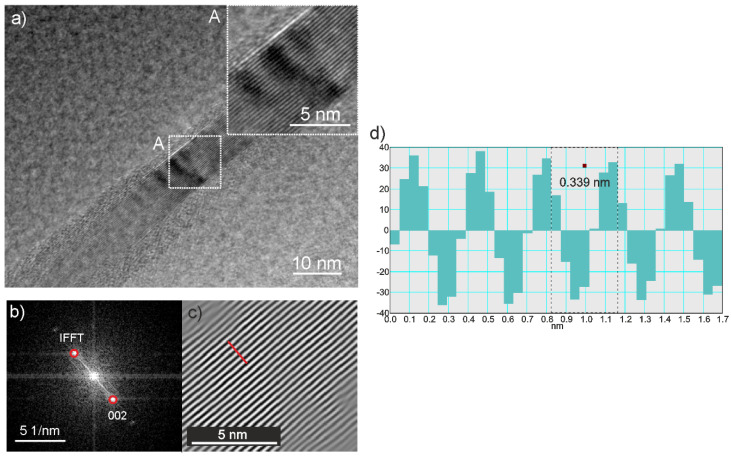
High resolution transmission electron microscopy (HRTEM) micrograph of graphene sheet with magnified details given at the top (**a**), fast Fourier transformation (FFT) patterns from the area marked as A in Figure 2a and its identification (**b**), inverse fast Fourier transformation (IFFT) image of the spot pairs marked by red circles in Figure 2b (**c**), and intensity profile for the line marked as red in its IFFT image showing its d-spacing as 0.339 nm (**d**).

Graphite particles used as a component of composite coatings were in the form of graphene packages with several layers (Figure 1a). The electron diffraction pattern deriving from the package showed a polycrystalline character (Figure 1b). The XRD pattern contained a strong crystalline diffraction peak from graphite (hexagonal primitive, hp) at the 2Θ angle of 26.4° (Figure 1c). The HRTEM image of a graphene sheet with a few layers clearly shows distinct lattice fringes (Figure 2a). The interplanar distance between the fringes measured in a fast Fourier transform (FFT) pattern (Figure 2b) and in a line profile pattern performed along the line marked in an inverse fast Fourier transform image (IFFT) (Figure 2c) of 0.339 nm, corresponded to the (002) plane of graphite with a hexagonal structure.

PEEK particles were thoroughly investigated in our previous work [20]. XRD studies showed an almost completely amorphous structure of the polymer powder with a trace amount of crystalline phase. The equivalent circle diameter of the particles was in the range of 2–15 μm.

Both in our previous works [20,45] and in the works of other authors [12,13,46], it has been proven many times that pure PEEK coatings can be successfully deposited on an anode from an ethanol-based suspension without any other additives. However, our experimental studies showed that the deposition of graphite from pure ethanol is impossible. According to the literature data [32,44], pure graphene coatings were successfully electrophoretically deposited from an isopropanol-based suspension. In those works, they firstly added graphene powder to isopropyl alcohol and then, after 1 h of ultrasonic dispersion, used magnesium nitrate (Mg(NO_3_)_2_ 6H_2_O) additive to charge the suspension.

In the case of composite coatings, interactions between the co-deposited particles in the dispersion phase greatly influence the final success of EPD. In our earlier work [21], we deposited a homogeneous PEEK-based coating containing Al_2_O_3_ from pure ethanol. However, there is much more frequently a situation in which it is necessary to use additional substances that will facilitate or even allow deposition. For example, Boccaccini et al. [16] and Seuss et al. [47] used citric acid monohydrate for the deposition of PEEK/bioglass coatings to stabilize the ethanol-based suspension. Baştan et al. [6] used HCl and NH_4_OH to obtain the specific pH of the suspension that prevented sedimentation during the deposition of polyetheretherketone and hydroxyapatite particles. In our previous works, we also used the addition of polyethylenimine or chitosan polyelectrolytes to ethanol for the co-deposition of PEEK with nitrides (Si_3_N_4_, TiN) [22,23,24] and sulfides (MoS_2_) [25]. Although the use of the polyelectrolytes made it possible to deposit macroscopically homogeneous coatings, unfortunately, the thermal degradation of PEI or chitosan during heat treatment after deposition resulted in the formation of open porosity in the coatings.

Because of the similarity in some respects, e.g., the form of thin platelets combined in packages or similar surface potential, it seemed that graphite particles may behave similarly to MoS_2_ nanosheets. Interestingly, in the present work, it turned out that, in contrast to MoS_2_/PEEK coatings, the graphite/PEEK coatings can be deposited from pure ethanol containing 1 g/L of graphite without the need for any additives. However, at higher graphite concentrations of 2 g/L and 4 g/L in ethanol, no deposition process was observed, regardless of the suspension’s pH, due to faster sedimentation of the suspension in comparison with the suspension with the graphite concentration of 1 g/L.

**Figure 3:**
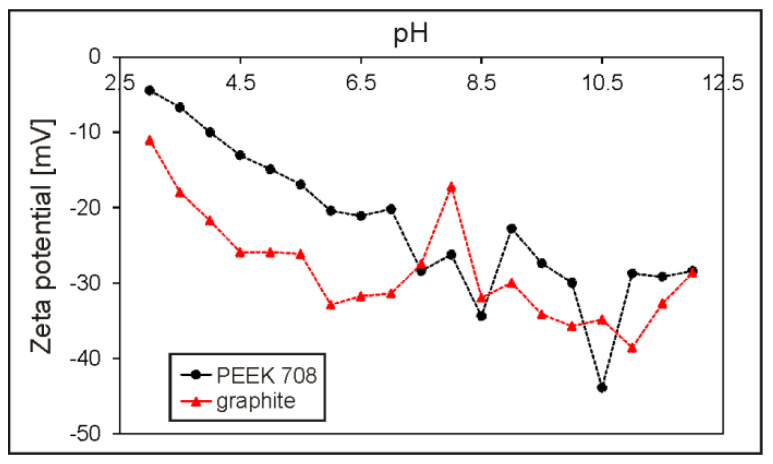
Zeta potential as a function of pH for graphite in ethanol and polyetheretherketone (PEEK) 708 in ethanol.

To understand and explain the phenomenon of deposition of PEEK and graphite from pure ethanol, zeta potential measurements and TEM observations of the suspension were performed. The zeta potential of both graphite and PEEK particles in ethanol was negative over the entire measured pH range, reaching the maximum values of –38.6 at pH = 11 and –43.9 at pH = 10.5, respectively (Figure 3).

**Figure 4:**
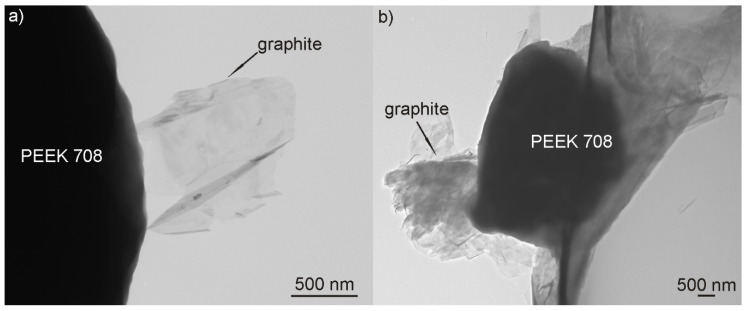
TEM images of the graphite particles adsorbed to the PEEK 708 microparticle in the suspension used for electrophoretic deposition (EPD) containing 1 g/L (**a**) and 2 g/L (**b**) of graphite. In the case of the suspension containing 1 g/L of graphite the PEEK particles are covered only partially.

**Figure 5:**
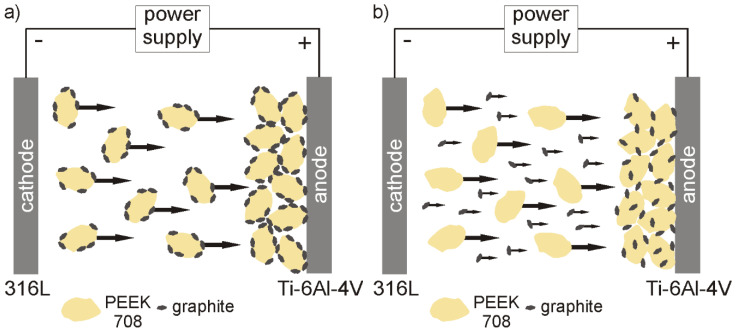
Schematic drawings of the possible co-deposition mechanism of the graphite and PEEK 708 particles from ethanol showing the adsorption of graphite on the PEEK surface and deposition of complexes on the anode (**a**), and independent deposition of both types of particles on the anode (**b**).

A sharp drop in the zeta potential value of the graphite particles to –17.2 mV was observed at pH = 8. It is supposed that this behavior may be due to the addition of citric acid to the ethanol in order to decrease the pH of the suspension from 8.7 (original pH) to 8, which provided citrate anions in the suspension and affected the electrophoretic mobility of graphite particles. Since the citrate anions were already present in the suspension, further acid addition did not cause such a significant change in zeta potential, which was in the range from –25.9 mV to –27.5 mV at pH = 4.5–7.5. However, at pH below 4.5, the zeta potential began to decrease gradually. The original, not adjusted pH of the suspension used for deposition was 8.7. Although the potential was not the highest at this point, it was quite sufficient for deposition because it oscillated around 30 mV for both particle types. According to the literature [48], this is the value used to assume that the suspension is relatively stable. TEM investigation of the suspension (containing 1 g/L of graphite) used for EPD revealed that the graphite particles were most often adsorbed on the surface of large PEEK particles and a representative image is shown in Figure 4a. It should be noted that, in this case, the PEEK particles were not completely covered with graphite. In contrast, it was observed that the PEEK particles in the suspension containing 2 g/L of graphite were completely or nearly completely covered with graphite particles (Figure 4b). Such behavior of particles indicates the electrostatic interactions between them and may explain the differences in the behavior of the particles during the EPD process, deposition and its absence, respectively, in relation to graphite concentration in the suspension. However, on the other hand, graphite particles and separate PEEK particles were also observed in the suspension. It should be mentioned that there are very large differences in both particle types, e.g., in nature, in size: micro and nano, in morphology: 3D and 2D, and in properties: hard and soft. However, regardless of these differences, the charge of both was interestingly the same. The electrostatic interaction occurs much more frequently between particles with opposite charges. For example, in the study of Yousefpour et al. [49], where negatively charged PTFE combined into composite particles with positively charged hydroxyapatite, or in the work of Castro et al. [50], where such a situation occurred between ZrO_2_ and MgO particles. However, a similar interaction between co-deposited particles to that observed in our study was described by Baştan et al. [6] based on the co-deposition of PEEK microparticles and hydroxyapatite nanoparticles. According to them, HA nanoparticles have higher specific surface energy, which causes interaction between HA and PEEK particles in the suspension. HA particles cover the surface of PEEK and form the PEEK/HA complex despite the fact that both particle types have a positive zeta potential. Based on the investigation results provided by zeta potential and TEM described above, it is possible to propose two deposition mechanisms, which may occur separately or together at the same time (Figure 5). They consist of (i) graphite adsorption on the PEEK surface and then complex particles (partially covered with graphite) deposit together on the positively charged electrode (anode) and (ii) independent deposition of both particle types on the anode. However, as confirmed experimentally, the deposition of graphite from pure ethanol is impossible, therefore the first mechanism is much more plausible.

The selection of the basic deposition parameters, voltage and time, was performed in an experimental way, and the preliminary evaluation of the coating homogeneity was carried out by macroscopic observations. The coatings were macroscopically homogeneous and repeatable after deposition from the suspension containing 1 g/L of graphite at pH = 8.7 in the voltage range from 50 V to 70 V. After deposition at voltages in the range of 20–40 V, coatings were too thin and did not cover the substrate completely, while the deposition at the highest voltages in the range of 80–100 V led to thick coatings being obtained, but they started to lose uniformity. It should be pointed out that the EPD process was also carried out from suspensions at lower pH after adding citric acid and at higher pH after adding sodium hydroxide. Specific suspensions were selected in which the zeta potential of graphite in ethanol was relatively high, i.e., at pH = 4.5, 6, 7, 9.5 and 11 (Figure 3). However, deposition was not observed at any of the following pH values. Moreover, faster sedimentation of the suspension was detected, compared to the virgin suspension without any additives. The zeta potential at pH = 8.7 was not the highest possible, but at the same time it was sufficient to allow deposition and good quality coatings to be obtained. In addition, the suspension used for deposition was environment-friendly pure ethanol and did not require any additional substances, such as acids or bases, which is an important advantage. Finally, the coating deposited at 70 V and 40 s was selected for further microstructural studies.

**Figure 6:**
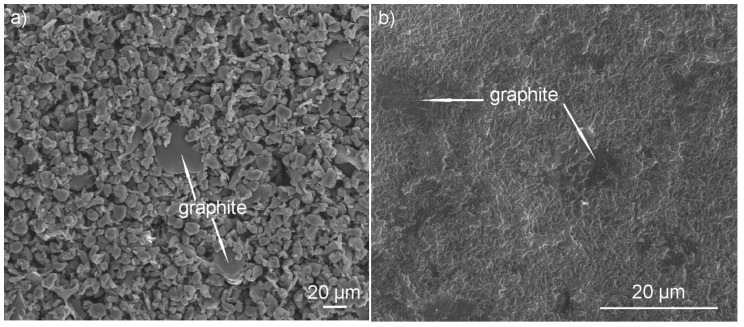
SEM images of the graphite/PEEK 708 as-deposited (**a**) and heat-treated (**b**) coatings.

Coatings after deposition and drying were submitted to heat treatment in order to densify them and to increase the adhesion to the substrate. Figure 6 shows SEM images of coatings directly after deposition and heat treatment. It can be seen from Figure 6a that the PEEK particles uniformly covered the substrate and between them the graphite particles were sporadically distributed. Figure 6b shows that the coatings after heat treatment were dense and no cracks or porosity occurred. Moreover, the PEEK changed its morphology to a continuous coating matrix, in which relatively evenly embedded graphite particles are visible on the coating surface. However, it can also be seen that the graphite content was rather low. A characteristic spherulitic microstructure can also be observed in the coating, which suggests the formation of a crystalline structure in the polymer during heat treatment. It should be noted that, according to the literature [20], a PEEK microstructure composed of spherulites is preferred in terms of tribological properties, because spherulites cause strengthening of the coating and reduce its destruction. It can be seen in Figure 6b that the graphite particles were oriented parallel to the coating surface, which is also preferable in terms of the subsequent tribological properties. The coating thickness was measured as 110 µm.

**Figure 7:**
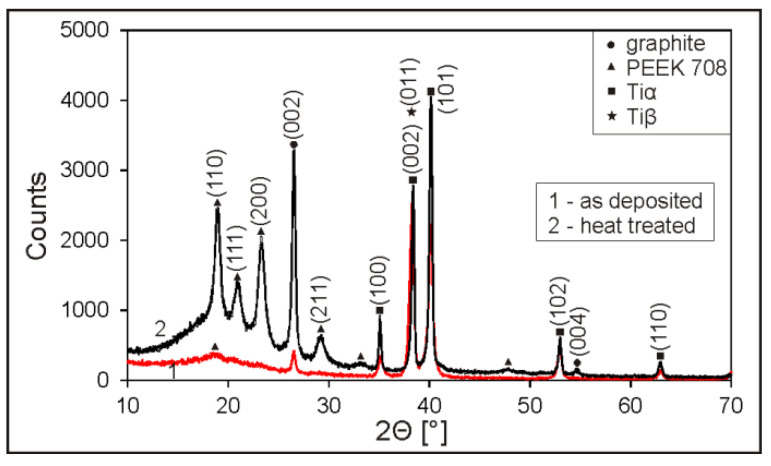
XRD patterns of the graphite/PEEK 708 as-deposited (1) and heat-treated (2) coatings on the Ti-6Al-4V alloy.

The XRD studies confirmed that PEEK after heating and cooling with a furnace changed its structure from amorphous to semi-crystalline (Figure 7). One wide amorphous peak from the polymer can be seen in the XRD pattern of the as-deposited coating, while four strong crystalline peaks are clearly visible in the pattern after the heat treatment of the sample. In both XRD patterns, before and after heat treatment, crystalline peaks from graphite at the 2Θ angles of 26.4° and 54.2° are also present.

**Figure 8:**
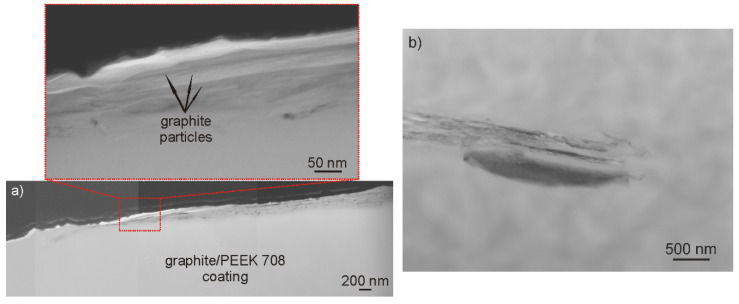
Microstructure of the outer area of the graphite/PEEK 708 coating heated at 390 °C and cooled with a furnace on a cross section. The graphite particle is located in the area directly close to the coating surface (**a**) and in the area about 3 µm away from the coating surface **(b**). Magnified details of the particle present in Figure 8a showing a layered structure are given at the top. TEM, focused ion beam (FIB) lamellae.

To investigate the coating microstructure on a cross-section by TEM, a lamella was cut out with the use of FIB. Similarly to the SEM study performed on plan-view samples, it was observed that the graphite particles consisted of graphene packages of 200 nm thick and oriented parallel to the coating surface occurred in the dense polymer matrix (Figure 8a). Only individual small graphite particles were visible in the deeper areas of the coating (Figure 8b).

**Figure 9:**
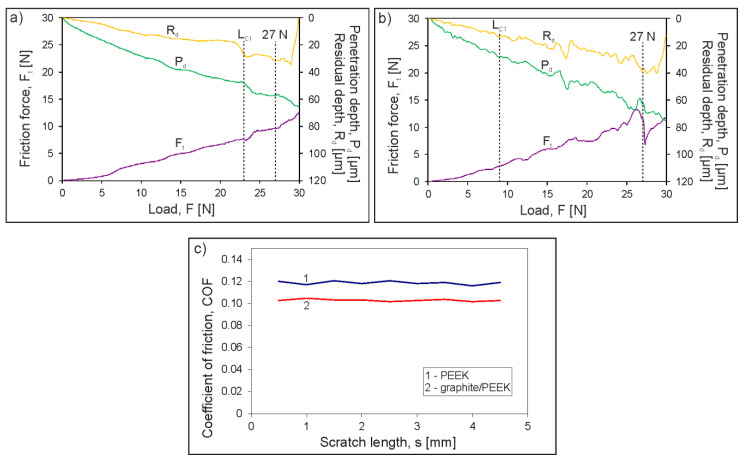
The scratch test results (friction force, penetration depth and residual depth signals) of PEEK coating (**a**), and graphite/PEEK coating (**b**), and friction coefficient of the coatings vs. diamond stylus in a scratch test at 0.25 N constant load (**c**).

**Figure 10:**
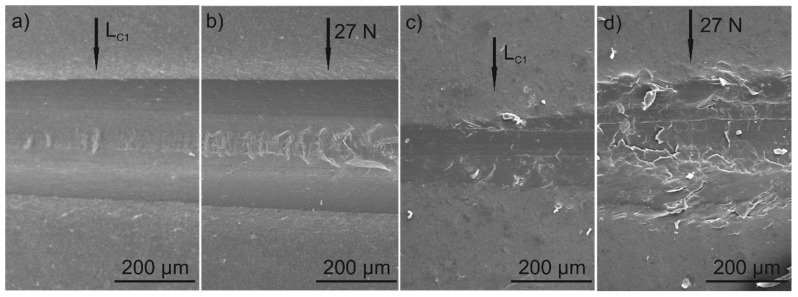
SEM images of the scratch track show the failure of PEEK coating at critical load L_C1_ = 23 N (**a**) and at 27 N (**b**) as well as graphite/PEEK coating at L_C1_ = 9 N (**c**) and at 27 N (**d**).

Scratch tests were carried out for both the unfilled PEEK coating and the graphite/PEEK coating under standard conditions with an increasing load of 0–30 N (Figure 9a,b) and at a constant load of 0.25 N (Figure 9c). The scratch tests results indicates very well adherence of the unfilled PEEK coating and graphite/PEEK composite coating to the substrate. Scratch with a Rockwell indenter did not cause any adhesive failure or delamination in any of the tested coatings, even under a load of 30 N (Figure 10b,d). However, the introduction of graphite fillers reduced the scratch resistance of the composite coating compared to the unfilled PEEK coating. The first single cohesive cracks in the graphite/PEEK coating were located in the middle of the scratch track at the load L_C1_ = 9 N (Figure 10c), while for the pure PEEK coating they were observed at the load L_C1_ = 23 N (Figure 10a). The number of cracks increased while increasing the load and they also occurred at the edges of the scratch tracks. At a load of 27 N, a small fragment of the graphite/PEEK coating surface was detached, but the substrate was not exposed (Figure 10d). Meanwhile, the failure of the pure PEEK coating at this load consisted only in the formation of more extensive and deeper cracks in the coating (Figure 10b). The explicit destruction of the coatings at the loads of L_C1_ and 27 N was reflected in the change of the course of friction force and the penetration depth (Figure 9a,b). It has been found that the graphite/PEEK coating is more highly susceptible to deformation than the pure PEEK coating. The penetration depth of the indenter (P_d_) at the maximum load was up to 75 µm and 65 µm for the composite and pure PEEK coatings, respectively, while, after unloading, the plastic deformation (R_d_) of both coatings in the scratch track was approx. 40 µm.

The beneficial effect of introducing the graphite filler into the PEEK polymer matrix manifested in the reduction of the COF of the graphite/PEEK coating during sliding contact with the diamond stylus compared to the pure PEEK coating. This was confirmed by the scratch tests carried out at a constant load of 0.25 N, which did not cause a large deformation of the polymer coatings, and thus the effect of the mechanical component of friction force on the COF value was minimal (Figure 9c).

**Figure 11:**
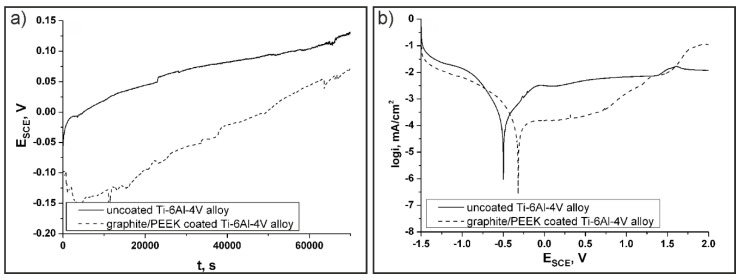
Electrochemical measurements of uncoated Ti-6Al-4V and graphite/PEEK coated Ti-6Al-4V alloy in 3.5 wt.% NaCl aqueous solution at 25 °C. Evolution of the corrosion potential vs. time (**a**) and polarization curves at 1 mV/s (**b**).

The open circuit potentials (OCP) were measured during a 20 h immersion test (Figure 11a). The potential value of the uncoated alloy at the beginning of the measurement equalled −0.05 V and changed in time, constantly increasing its value until it reached about 0.11 V after approx. 50,000 s. Similar plots, but with slightly lower values of potentials (−0.10–0.05 V), were observed for the graphite/PEEK coated alloy. Our previous study [20] of pure PEEK coatings indicated comparable OCP results for uncoated and semi-crystalline PEEK coated titanium alloys. In Figure 11a, the differences between OCP at the start and after approx. 50,000 s of measurement were significantly smaller. Because of the very small distance between OCP results for uncoated and graphite/PEEK coated alloys, it can be said that the corrosion resistance was similar for both the uncoated and coated titanium alloy. The anodic cyclic potentiodynamic polarization curves of both samples in sodium chloride solutions are presented in Figure 11b. A passive behaviour with large passive potential range resulted for both uncoated and coated alloy. The corrosion was defined by the limiting current density, which passes through the passivating film, thus becoming a measure of the film’s protective performance [51]. The passive current density (i_p_) was reduced from 5 µA/cm^2^ for the uncoated sample to 0.33 µA/cm^2^ for the coated ones, while the corrosion potential (E_corr_) increased from ~ −0.50 to −0.32 V, respectively. This shift in the polarization curve indicates improved corrosion resistance of the coated alloy as the coating acts as a protective layer. According to our previous work [20], semi-crystalline PEEK coatings on the Ti-6Al-4V substrate had quite similar results of polarization tests.

These studies showed that EPD is flexible enough to fabricate PEEK-based coatings incorporated with graphite particles, adheres well to the titanium alloy substrate and exhibits a tailored microstructure consisting of 2D graphite distributed in the coating areas close to the surface and oriented parallel to the surface. In our previous studies, we clearly demonstrated that the PEEK-based coatings containing hard Al_2_O_3_ [21] or Si_3_N_4_ [22] or TiN [24] nanoparticles significantly improved the wear resistance of the Ti-6Al-4V titanium alloy in dry sliding contact with an alumina ball. The wear index was 0.47 10^−6^, 1.41 10^−6^ and 1.10 10^−6^ [mm^3^/Nm], respectively, after the same ball-on-disc test conditions. In comparison with the pure PEEK708 coating, the wear rate was higher (2.61 × 10^−6^ [mm^3^/Nm]) during the same test conditions. However, the COF was still relatively high and equalled 0.27 for the pure PEEK708 coating [20], 0.25 for the Al_2_O_3_/PEEK708 coating [21], 0.26 for the Si_3_N_4_/PEEK 708 coating [22] and 0.30 for the TiN/PEEK 708 coating [24]. Similarly, in other works on PEEK and PEEK-based coatings, a relatively high coefficient of friction was demonstrated. For example, Li et al. [52] reported that the COF of PEEK coatings in cooperation with a 100C6 steel ball was around 0.3. Zhang et al. [53] also received a COF of PEEK and SiC/PEEK coatings of approximately 0.3 and 0.4, respectively, in cooperation with a 100C6 steel ball. Thus, in the search for new advanced PEEK-based coatings with a good balance between wear resistance and a low COF, the successful co-deposition of PEEK and graphite with a tailored microstructure is very promising. However, from the point of view of developing low-friction coatings, it seems advisable to increase the content of graphite, as a lubricant phase, in the coating. Therefore, future work will concentrate on the optimization of the PEEK to graphite volume fraction to obtain coatings with a good balance of wear resistance and COF as well as electrochemical corrosion resistance.

## 4. Conclusions

1. Nanocomposite graphite/PEEK 708 coatings were electrophoretically deposited on titanium alloy substrates from an environmentally-friendly ethanol-based suspension containing 1 g/L of graphite. The optimal parameters to obtain homogeneous coatings were: voltage of 70 V and deposition time of 40 s. The EPD mechanism was investigated and discussed. The most probable mechanism of co-deposition of the particles consisted of the electrostatic interaction between them. As a result, graphite particles adsorbed on the surface of PEEK microparticles and graphite-PEEK complexes were deposited on the anode.
2. The post-EPD heat treatment densified the coatings and increased their adhesion to the titanium alloy substrates. The coating had high scratch resistance and there was no adhesive damage. The first cohesive cracks appeared at the load L_C1_ = 9 N and their number grew with increasing load. The coating was characterized by high susceptibility to deformation, and plastic deformation of the coating after unloading was up to 40 μm.
3. As a result of duplex treatment, the graphite particles embedded in the polymer were oriented parallel to the coating surface, which is the most advantageous arrangement in the context of friction processes. However, their amount was rather low and the further optimization of their content in the coating to achieve a stable and low COF is necessary.
4. The coated alloy exhibited better corrosion resistance compared to the uncoated alloy in a sodium chloride solution at a temperature of 25 °C.

This study showed that a combination of electrophoretic deposition and post-EPD heat treatment is a convenient way of obtaining composite PEEK-based coatings incorporated with graphite particles consisting of graphene layer stacks oriented parallel to the coating surface. Such coatings are an important subject of research to improve the tribological properties of titanium alloys, while increasing frictional wear resistance and lowering the friction coefficient.
